# Current oral contraceptive use affects explicit and implicit measures of depression in women

**DOI:** 10.3389/fpsyg.2024.1462891

**Published:** 2024-10-18

**Authors:** Elizabeth Hampson, Sara N. Abrahamson, Taylor N. Breddy, Maisha Iqbal, Elena R. Wolff

**Affiliations:** ^1^Laboratory of Neuroendocrinology, Department of Psychology, University of Western Ontario, London, ON, Canada; ^2^Neuroscience Program, Schulich School of Medicine & Dentistry, University of Western Ontario, London, ON, Canada

**Keywords:** oral contraceptive, hormonal contraceptive, mood, affect, depression, depressive, implicit, progestin

## Abstract

Some data suggest that increased depressive symptoms may occur in women using combined oral contraceptives (OCs). However, this idea is controversial and the existing evidence is conflicting. The present study compared negative affect in 53 healthy women (*M*_age_ = 19.9 years) during intervals of active daily OC hormone intake and during the washout week of the contraceptive cycle when no exogenous estrogens or progestins are used. A prospective counterbalanced repeated-measures study design was employed. Depressive affect was evaluated using standard psychometric tests of explicit (self-perceived) and implicit negative affect. Implicit measures are considered less subject to bias related to social expectations, self-awareness, or willingness to disclose. Other than their usual OCs, participants were medication-free and had been using OCs for a median of 12 mo. We found that measures of implicit affect (e.g., Affect Misattribution Procedure, Emotional Stroop Test) displayed a more depressive-like pattern of performance during active hormone intake, particularly among a subgroup of OC users who reported experiencing high levels of depressive affect more generally. In contrast, participants’ self-perceptions suggested that they perceived their negative symptoms to be greater during the ‘off’ phase of the OC cycle, when OC steroids are withdrawn and menses occurs. The present findings reinforce the possibility of depressive mood effects associated with OC usage, and highlight the utility of including implicit measures, but also illustrate the complexity of mood assessment in OC users.

## Introduction

Oral contraceptives (OCs) are used by over 150 million women worldwide (United Nations Population Division, 2019). Most OCs are ‘combined’ oral contraceptives consisting of 2 hormonal constituents: a synthetic estrogen and a progestin. Individual brands vary in dose and the exact progestin used, but all OCs inhibit ovulation and thus reduce the possibility of conception (Dickey and Seymour, 2021). While their gynecological actions are well-documented, little is known about OC effects in the central nervous system (CNS). Animal studies have indicated that estrogen and progesterone receptors are widely distributed in the CNS (Brinton et al., 2008; Östlund et al., 2003; Weiser et al., 2008), and hormone-receptor binding can produce a wide spectrum of regional changes in neurotransmission and synaptic plasticity (Barth et al., 2015; Galea et al., 2017). The synthetic hormones used in OCs can bind to these receptors with affinities that may equal or sometimes even exceed their endogenous ligands (Escande et al., 2006; see Hampson, 2023 for an overview). Despite this fact, scientific knowledge of OC effects in the CNS is still rudimentary.

Although high in contraceptive efficacy, a frequent reason why women discontinue their use of OCs is self-perceived mood changes (Bancroft and Sartorius, 1990; Lundin et al., 2017; Rosenberg and Waugh, 1998). These include increased irritability, mood lability, or depressive affect. Although reported as far back as the 1970s, most evidence for depressive mood changes among OC users has been anecdotal or based on self-reports. Over the past 8–10 years an upsurge in research in the neuroscience and epidemiology communities has sought to document the occurrence, prevalence, scope, and severity of OC-related mood effects, including a number of large-scale population-level studies (e.g., Gawronska et al., 2024; Johansson et al., 2023; Skovlund et al., 2016; Toffol et al., 2011; Wiréhn et al., 2010; Zettermark et al., 2018). However, the findings have been mixed and conflicting. Several recent large-scale studies confirmed an increase in rates of clinical depression among OC users relative to non-users (e.g., Skovlund et al., 2016; see also Skovlund et al., 2018) based on markers such as antidepressant use, suicidal behavior, or documented clinical diagnoses, but other work also using large samples has failed to confirm any differences (McKetta and Keyes, 2019; Lundin et al., 2022) or, less frequently, even suggested a protective effect of hormonal contraception (Keyes et al., 2013; Toffol et al., 2011). Some data suggest adverse mood effects may be limited to adolescents or those who initiate OC use during adolescence (Anderl et al., 2020; de Wit et al., 2020; Johansson et al., 2023; Zettermark et al., 2018). Yet other studies have found that adult women who otherwise experience premenstrual dysphoria when in a non-medicated state may in fact experience greater stabilization in mood under OC use (Bäckström et al., 1992; Lundin et al., 2017). Studies addressing potential CNS mechanisms for depressive changes are even fewer in number (e.g., Larsen et al., 2020; Larsen et al., 2022; Porcu et al., 2019) and are at present, inconclusive. Consequently, the question of negative mood change under OC use remains unanswered.

Even among those studies that report increased rates of depression, the numbers of women who experience clinical depression under OC use is small (an estimated 4–10%; Skovlund et al., 2016; Hampson, 2023; Sundström Poromaa and Segebladh, 2012). This is far below the self-reported rates of mood change among OC users, which range as high as 30–50% (Grant and Pryse-Davies, 1968) and might reflect the magnitude of the changes. We propose that larger numbers of women may experience subclinical increases in dysphoria which are milder and thus not identified by recent clinical studies that have focused on formal clinical diagnoses of major depressive disorder and/or the use of antidepressant therapies. Indeed, a historical review of the question of mood change in OC users (Oinonen and Mazmanian, 2002) advocated for the use of a dimensional approach to evaluate mood as key to future progress. The possibility of subclinical mood disturbance has received little systematic research to date, and is thus a significant gap in developing a complete understanding of the spectrum of OC effects on emotional processing. Factors that determine whether mood changes occur under OC use, and their severity, likely include both person-specific vulnerabilities and drug-related variables such as differences in dose, type of progestin, or duration of OC use, all of which have a potential to contribute to variability in outcomes. Using a dimensional approach, we recently found that negative affect varied by OC progestin subtype in a sample of 193 long-term OC users taking various standard OC drug formulations (Hampson, 2023). In general, the phenomenon and scope of mood changes in response to OC initiation has been inadequately explored. Developing an improved understanding is important because of the significance of mood status for women’s quality of life and interpersonal functioning (including their perceptions of, and interactions with, significant others in their social environment).

In the present pilot study, a repeated-measures design was used to evaluate affective status in female users of OCs. We employed a formal self-report measure of depression and other dimensions of mood, but we also used implicit measures, in which depressive affect manifests itself automatically, without overt participant awareness, via mood-driven changes in accuracy or speed of responding to affectively-laden visual stimuli. To our knowledge, the present work is the first to include implicit measures to detect depressive affect in OC users. Implicit measures are sometimes considered a ‘truer’ window into the affective system, as responses are rapid and driven by automatic associative learning processes that operate outside the direct awareness of participants and are thus less subject to reporting or disclosure biases that can impede explicit, deliberate, self-reports (Suslow et al., 2019; DeCoster et al., 2006). Though not used previously to study OC users, implicit tasks have been used in past studies of major depression in other contexts, where they have revealed selective differences in the emotional processing characteristics of depressed versus non-depressed individuals (Leppänen, 2006; Weightman et al., 2014). Implicit affectivity is also predictive of spontaneous behavioral, autonomic, and HPA (hypothalamic–pituitary–adrenal) responses to everyday emotional stimuli and stress (e.g., Bodenschatz et al., 2018; van der Ploeg et al., 2016; Quirin et al., 2009).

In the present work, our major focus was users’ perceptions and reactions to facial expressions of emotion. If hormones in OCs do engender a more depressive mindset, we expected to find higher depression scores on an explicit measure of mood during the ‘active’ phase of the contraceptive cycle (when synthetic hormones are actively used each day) than during the monthly ‘inactive’ phase when hormones are not used. We also expected to find a more depressive profile of responses on implicit measures during active intake.

## Materials and methods

### Participants

Participants were 62 female university students or administrative staff ages 18–26 years, who had used a standard combined OC for at least the past 3 months (median = 12.0 months) and had no chronic health conditions or other prescription medications. One volunteer diagnosed recently with depression was retained in the sample. Demographics are given in Table 1. Participants were recruited through flyers at the university and were reimbursed a total of $25 to cover their travel costs.

**Table 1:** Participant Demographics (*N* = 62).

Mean age at testing (years)	19.92 (*SD* = 1.85)
Mean age at first OC use (years)	17.45 (*SD* = 1.74)
Mean years of university study	2.55 (*SD* = 1.55)
Median time on current OC pill (months)	12.00
Type of progestin used:	
Norethindrone acetate	*n* = 4
Levonorgestrel	*n* = 26
Norgestimate or desogestrel	*n* = 21
Drospirenone or cyproterone	*n* = 11
	Total = 62
Current brand of OC:	
Number of monophasic brands (%)	54 (87%)
Number bi- or triphasic brands (%)	8 (12%)

OC, oral contraceptive.

### General procedure

A repeated-measures design was used. Two counterbalanced test sessions were scheduled, and were performed during the active intake of OC hormones (“Active”) and during no hormone intake (“Inactive”). Although infrequently used to study OC effects, this type of study design can identify short-term effects of OC use that depend on changes in the serum availability of exogenous steroids. Such designs have been used previously to identify cognitive or affective alterations associated with active intake of OCs (e.g., Noachtar et al., 2023; Hampson et al., 2022) and are especially powerful if used in a within-subjects context as recommended by several recent reviews (Beltz, 2022; Hampson et al., 2022).

The active test session took place during the second or third week of the active phase of each woman’s contraceptive cycle (i.e., after 14–21 consecutive days of daily ethinyl estradiol and progestin use). Timing of the testing was controlled and coincided with the days of maximum OC steroid dosage based on the particular brand of OC pill used by each individual. Thus the timing was individualized to each participant’s OC regimen, based on contraceptive pill and health information shared in advance via a brief online screening. The Inactive session was also tightly controlled. It took place during the ‘washout’ or ‘inactive’ days of the cycle, after at least 3 days of no hormone use (*M* = 4.81 days after the last active pill was taken) to allow adequate washout of the OC steroids to occur. Once again, the exact timing was personalized and was dictated by each individual’s specific brand of OC.

On the test day, participants reported to a university laboratory for a 40–45 min test session where explicit and implicit measures of mood were administered one-on-one by a trained examiner. Although the timing of each session was targeted prospectively to coincide with specific timepoints in the OC cycle, we also monitored and verified the timing retrospectively. Specifically, participants brought their current package of OC pills to each study visit to allow the researchers to visually inspect and verify the exact prescription details, brand name of OC pill, and the number of pills used (or still remaining) on the date of testing, to confirm that the desired timepoints had been properly targeted (or detect any scheduling errors). In addition to assessment of mood, participants completed several control tasks during each session and provided details about their past and present OC use. The study was approved by the University of Western Ontario Non-Medical Research Ethics Board and was performed in compliance with the Declaration of Helsinki.

### Explicit and implicit tasks

#### Profile of Mood States (POMS)

The POMS (McNair et al., 1992) was given at the beginning of each session before any other task. It is a widely used, extensively standardized, well-validated 65-item psychometric mood scale suitable for assessment of both clinical/psychiatric and non-clinical populations. It was considered an explicit measure of mood because respondents must reflect and self-assess their own emotional state. For each of 65 mood descriptors (e.g., ‘happy’, ‘worthless’, and ‘grouchy’), participants rated how intensely they felt that way over the past week using a Likert scale that ranged from 0 (*Not at All*) to 4 (*Extremely*). Total scores for 6 factor analytically-derived mood dimensions can be computed (Anxiety, Depression, Anger/Irritability, Vigor, Fatigue, and Confusion). Only the Anxiety, Depression, Anger, and Vigor subscales were relevant to the present hypotheses and were analyzed here.

Details of procedures for all tasks are described in the Supplementary material.

#### Facial Emotion Identification Task (FEIT)

This task was used to evaluate the emotional decoding of faces. Stimuli consisted of 96 images of adult faces expressing six basic emotions (Happy, Sad, Anger, Fear, Disgust, and Neutral), selected from the Pictures of Facial Affect (Ekman and Friesen, 1976) or the Racially Diverse Affective Expression (RADIATE) database (Conley et al., 2018; Tottenham et al., 2009). Stimuli were presented by computer in a random sequence using E-Prime 3.0 software (Psychology Software Tools, Sharpsburg, PA). Each image was a full-frontal view of a face.

Following 4 practice trials, 96 test faces were presented one at a time. Participants were asked to identify each emotional expression by pressing a button as soon as it was recognized. Response time (RT) was measured in milliseconds (ms) from image onset to keypress response. Participants also rated the perceived intensity of each image on a 10-point scale. Past work suggests that intensities are perceived to be more intense in the presence of depression (Weightman et al., 2014). On FEIT tasks, accuracy of identification is typically at or near ceiling in healthy individuals if an open-ended exposure duration is used (e.g., Hampson et al., 2006). Because our intent was to use the FEIT to evaluate implicit not explicit processing, the dependent variable used for analysis was the median time required for each participant to correctly recognize each of the 6 emotions at the active phase versus inactive phase of the OC cycle. Medians were used instead of means to limit the influence of occasional outlier trials where a participant had an unusually long (or short) RT. Previous literature shows that negative stimuli, such as sad or angry faces, capture visual attention more readily in depressed individuals than non-depressed and elicit greater difficulty in attentional disengagement (for reviews see LeMoult and Gotlib, 2019; Leppänen, 2006; Phillips et al., 2010; Ros et al., 2023). This ‘negativity bias’ reflects the greater attentional salience of negative stimuli in major depression, which is seen for faces but is also seen for negative stimuli or events more broadly (LeMoult and Gotlib, 2019).

#### Affect Misattribution Procedure (AMP)

**Figure 1:**
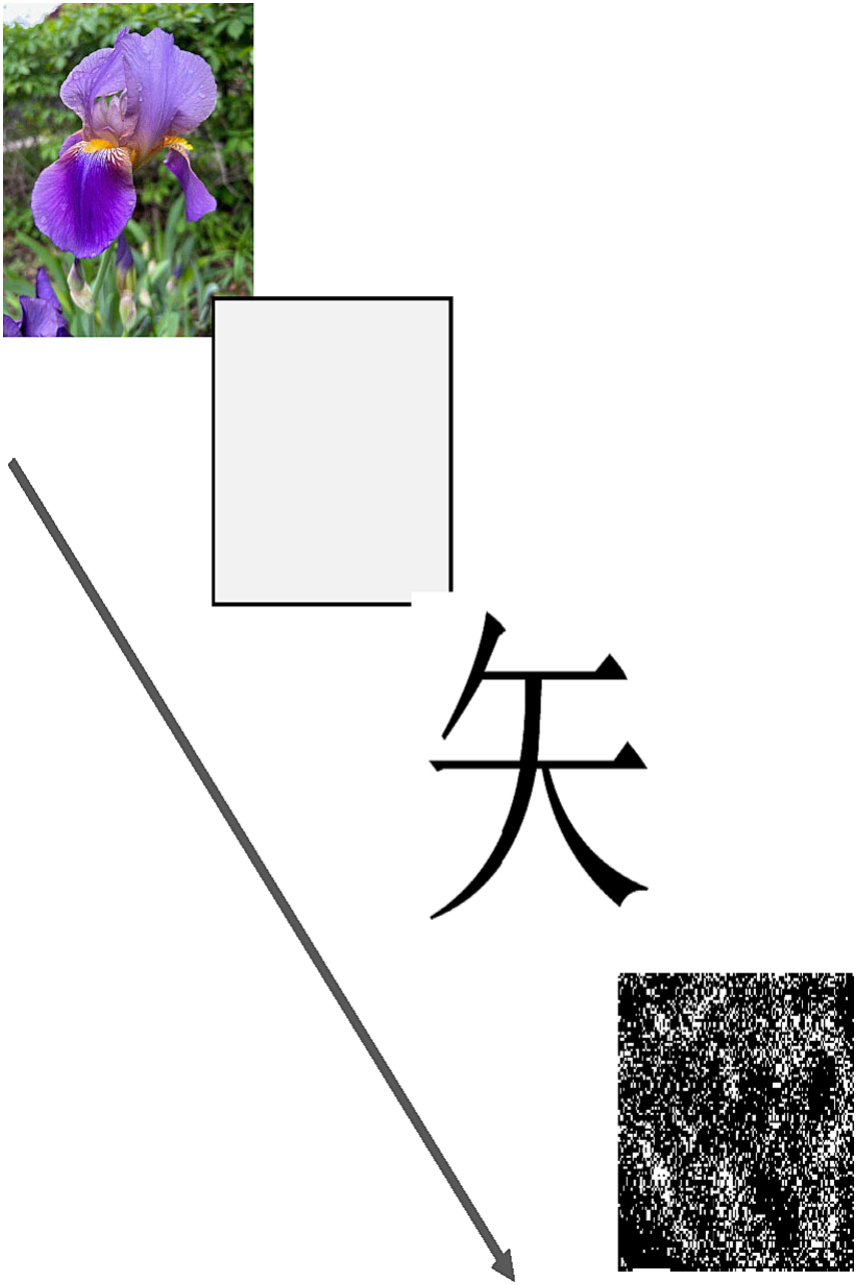
The sequence of events seen on each trial of the AMP (Affect Misattribution Procedure). The priming stimulus (a flower in this example) appeared first, followed by a randomly selected Chinese character. A visual mask then appeared, which served to terminate visual processing via backward masking. The arrow indicates the timeline and order of events on each trial.

The AMP (Payne et al., 2005) is a well-established implicit task. In the classic AMP, a sequence of affectively neutral visual stimuli (unfamiliar Chinese characters) is presented rapidly on a computer screen. Participants are asked to classify each target as aesthetically pleasing or unpleasing by pressing a response key as each character is shown. If a stimulus is preceded by an image of a flower or insect presented at an extremely short or even imperceptible duration, the percentage of the neutral stimuli classified as pleasant or unpleasant is biased by the priming stimulus toward a more pleasant (for flowers) or unpleasant (for insects) classification, instead of the 50% expected by chance. In the present work, the classic AMP was used to test for any difference in AMP scores between the active and inactive sessions, which might indicate a covert change in underlying affective processing. A randomized set of 48 images was presented via an Inquisit software script (Inquisit Lab 5, Millisecond Software LLC, Seattle WA; see Figure 1). The percentage of targets judged as pleasant when preceded by a flower, and when preceded by an insect, was tabulated.

To look more directly at perceptions of facial emotion, we also created an identical AMP task that exactly paralleled the classic AMP but where emotional faces (happy, and sad or angry) were used as primes instead of flowers and insects. Consistent with the hypothesis of greater depressive affect during OC usage, we predicted that positive stimuli would be less effective as primes when women were evaluated during active OC use, and that this would be true for both the classic and face versions of the AMP. Negative primes were expected to capture attention more strongly during active use, but we did not make a directional prediction for the negative stimuli due to conceptual ambiguity as to whether greater capture would be expected to increase or decrease transfer to the Chinese character that followed.

#### Emotional Stroop

An Emotional Stroop task was our final measure of implicit processing. We used a task previously described by Başgöze et al. (2015) in a study of major depression. Stimuli were presented and response times recorded (ms) using E-Prime 3.0. Briefly, a randomized series of faces from the RADIATE set were presented on a computer screen at a short duration (max 1,000 ms). Each compound stimulus (*N* = 128 trials) consisted of an adult face in the background (happy or else angry/sad) on which an English word printed in 60-point font (e.g., “harmony”) was super-imposed. The word was positioned at about mid-face. Participants were instructed to classify, as rapidly as possible, each word as having a positive or negative meaning, while ignoring the face in the background (which was irrelevant to word classification).

Emotional faces receive implicit processing if present, even if they are to be ignored or are irrelevant to the task at hand (e.g., Strand et al., 2013). In healthy controls, emotional faces typically interfere with the processing of words if the faces and words are of an incongruent valence (Strand et al., 2013; Başgöze et al., 2015). The interference effect is manifested as slower response times and/or increased word classification errors on the incongruent trials. Individuals with clinical depression show an interference effect, but display longer processing latencies (e.g., Epp et al., 2012) and an interference effect that may be amplified (see Epp et al., 2012 for a review; Ros et al., 2023). Some investigators, however, argue that the increased attentional salience of negative stimuli in people with depression can erode the ability of a conflicting positive word to generate a Stroop interference effect (e.g., Başgöze et al., 2015; Hu et al., 2012). If OC hormones are associated with increased dysphoria, we predicted that a larger Stroop effect would be found in the active condition where hormones are actively used, compared with the inactive condition where OC use is absent.

To rule out the possibility that any observed changes in the Emotional Stroop reflected, instead, an alteration in inhibitory control associated with OC use, rather than a change in affective processing, we gave a conventional non-affective Color-Word Stroop task to all participants as a control task. Only colors and words were used as stimuli; no affective processing was involved (Supplementary material).

### Statistical analysis

Data were analyzed using IBM SPSS Statistics 29.0. Repeated-measures ANOVA was employed to analyze each task, with OC Intake (Active, Inactive) as a within-subjects factor. For tasks with more than one condition (e.g., subscales of the POMS), Condition served as a second within-subjects factor. As described further below, depressive status was a between-subjects factor in the ANOVAs for the implicit tasks, with *post hoc* simple effects analysis where relevant, to test for an influence of depressive affect on the implicit scores. Bonferroni correction was used to evaluate significance for the explicit measure (the POMS). Because the present study is the first of its kind, performance on the implicit tests was evaluated at the *α* = 0.05 threshold for significance in order to minimize Type II error. Partial eta squared was used to quantify effect sizes.

The dataset from this study is openly available from the Open Science Framework (Hampson et al., 2024, http://doi.org/10.17605/OSF.IO/WCRN4).

## Results

Of the original 62 participants who enrolled in the study, 6 had valid data for one session only, due to scheduling error (*n* = 3) or failure to return for the second assessment (*n* = 3). In addition, 3 used Lolo, an atypical OC that has an inactive interval just 2 days long (limiting metabolic decline in the exogenous hormones that would otherwise occur during the inactive phase, Edelman et al., 2014; Dickey and Seymour, 2021). Because our hypotheses assumed decreased hormone concentrations are present at that time, Lolo did not afford a valid test of our study hypotheses. Thus, the final sample size available for statistical analysis was 53.

Of note, all 3 of the women lost to follow-up prior to their second testing showed elevated POMS depression scores at their initial assessment (*M* = 28.67), relative to population norms for their age and biological sex.

### Profile of Mood States

**Figure 2:**
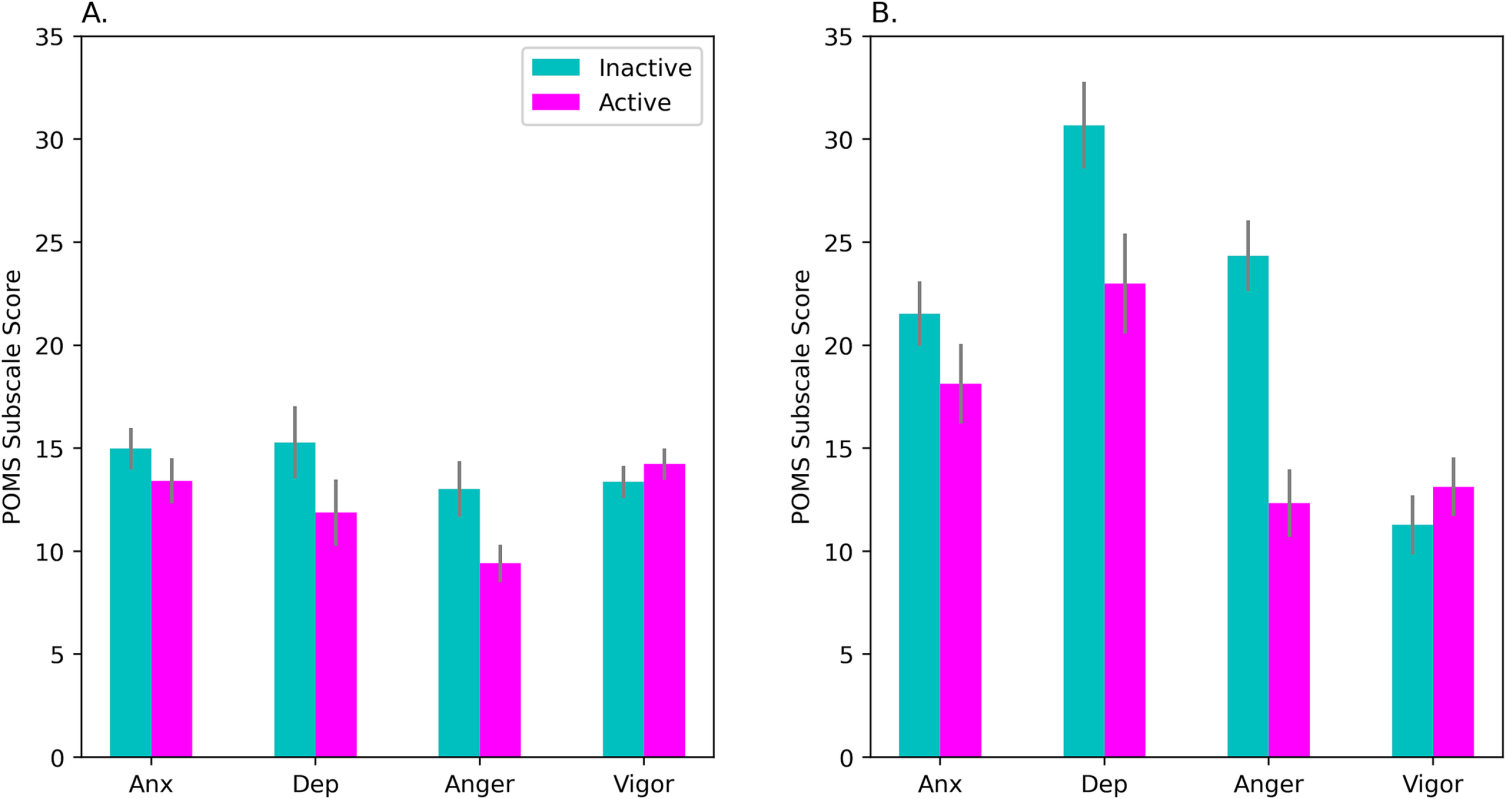
Mean scores on the POMS during days of active hormone intake (‘Active’) and no-intake (‘Inactive’). In **(A)**, results are shown for our sample of OC-users as a whole (*N* = 53). Panel **(B)** shows results for the HiD subgroup (*N* = 15) who had elevated self-reported Depression scores that matched levels usually observed in clinical patients with mild to moderate depression. Scores on the Anger and Depression subscales varied with OC intake (Bonferroni correction). The intake effect was seen for the OC-users as a whole, but was significantly larger in the HiD-subgroup, as seen in **(B)**. On the Anxiety subscale, the effect of OC intake was marginally significant (*p* = 0.057; see panel **A**). POMS, Profile of Mood States; Anx, anxiety subscale; Dep, depression subscale; Anger, anger subscale; Vigor, vigor subscale. Bars show the standard error of the mean.

Self-evaluated mood was analyzed via repeated-measures ANOVA with OC Intake (Active, Inactive) and POMS Subscale (Anxiety, Depression, Anger, and Vigor) as factors. The raw data were log-transformed before analysis to correct skewness on 2 of the subscales. However, in Figure 2 raw means are shown to permit easier comparisons with past literature.

Mean scores on all POMS subscales, including Depression, fell within established norms for female college/university students ages 20–21 (McNair and Heuchert, 2010, *N* = 516). However, this masked considerable within-group variability in our sample. ANOVA showed a significant OC Intake × Subscale interaction, *F*(2.19, 113.64) = 4.66, *p* = 0.009; *η*^2^_p_ = 0.082, with Huynh-Feldt correction (see Figure 2A). Contrary to *a priori* prediction, negative affect was higher during the inactive not active interval. Differences on the Depression (*p* = 0.009) and Anger (*p* = 0.005) subscales were significant by *post hoc* test (after Bonferroni correction, see Figure 2A).

Importantly, a subset of the women (29% of our sample of 62) showed substantially elevated POMS Depression during Session1 (*M* = 27.22, *SD* = 13.22) and/or Session2 (*M* = 26.73, *SD* = 14.57). In this subgroup, total Depression scores reached a magnitude commonly seen in outpatients diagnosed with anxiety or mild to moderate depression (*M* = 28.0, *SD* = 15.9, *N* = 650 adult female outpatients), according to the clinical test norms of the POMS (McNair and Heuchert, 2010). Below, we refer to this subset as the “HiD-subgroup.”

An exploratory ANOVA taking Subgroup into account confirmed greater negative affect in the HiD-subgroup (see Figure 2B), but also revealed that effects of OC intake on mood were accentuated in the HiD women compared with Non-HiD, OC Intake × Subscale × Subgroup interaction: *F*(1.75, 89.19) = 6.85, *p* = 0.003; *η*^2^_p_ = 0.118. In the Non-HiD subgroup the same pattern of means was observed, but more weakly. However, in the Non-HiD women the Depression subscale nevertheless exhibited a significant OC Intake effect (*p* = 0.038).

### Facial Emotion Identification Test

Accuracy on the FEIT was at or close to ceiling for several of the emotions we tested. These high accuracies indicate careful responding throughout the task. This was achieved via participants self-adjusting their response latencies (RTs) to maintain accuracy. Consequently, response latency was considered a superior indicator of any alterations in emotional processing that may be present.

Only the RTs were examined statistically. Data were analyzed using a three-way ANOVA with OC Intake (Active, Inactive), and Emotion (Disgust, Sad, Fear, Anger, Happy) as within-subjects factors and Subgroup (HiD, Non-HiD) as a between-subjects factor. The data were screened for outliers prior to analysis, and two outliers with RTs >3SD above the mean for their group were removed.

**Figure 3:**
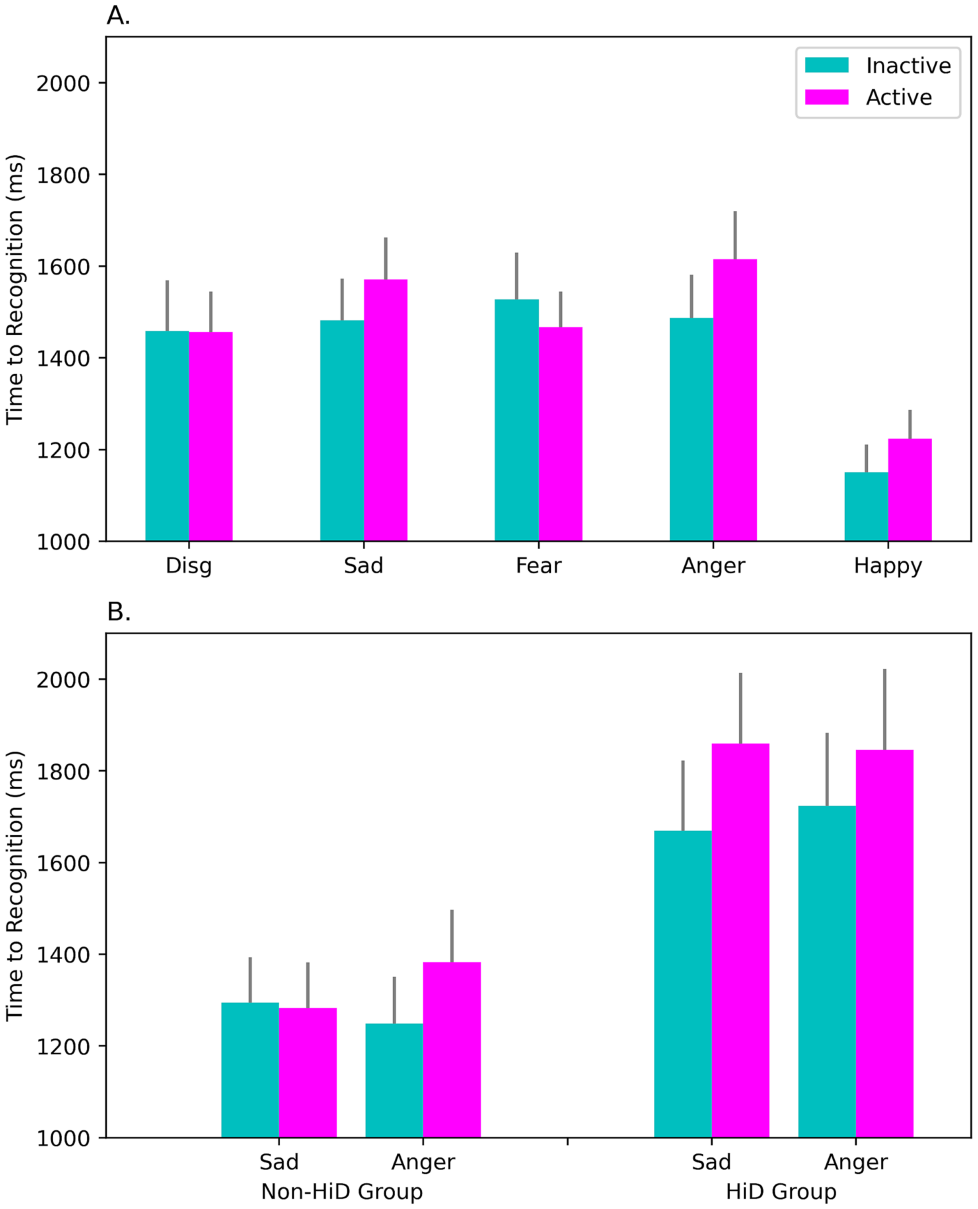
Response times (RT) to emotional faces on the FEIT were slower during active OC intake (‘Active’) than during the inactive interval (‘Inactive’). Panel **(A)** shows mean RTs in the sample as a whole (*N* = 51), for each facial emotion we assessed (disgust, sadness, fear, anger, and happiness). RTs were slower during active intake, and this was most evident for the Sad and Angry faces, whereas fewer attentional resources were allocated to the processing of happy expressions (which showed a more rapid RT response). Slowing was greatest among women in the HiD-group (rightmost bars in panel **B**), who reported high levels of negative (depressed) affect. Significant slowing was observed at both phases in the HiD women but was especially marked during the active intake of OC steroids. Slowing may signify the capture of attention by negative emotional stimuli relevant to depression and/or may indicate greater difficulty in recognizing the emotions displayed. FEIT = Facial Emotion Identification Task.

Responses were noticeably slower (longer RT) in the HiD-subgroup, consistent with past work on depressive disorder, main effect of Subgroup: *F*(1, 49) = 7.26, *p* = 0.010; *η*^2^_p_ = 0.129; mean for HiD = 1651.33 ms, mean for Non-HiD = 1236.15 ms. Importantly, in the sample of OC users as a whole (see Figure 3A), active OC intake was associated with slower RTs than inactive, particularly for certain key emotions, Emotion × Intake interaction: *F*(4, 196) = 4.29, *p* = 0.002; *η*^2^_p_ = 0.081. As seen in Figure 3A, slowing was clearest for sad and angry expressions, consistent with greater attentional capture reported in depressed individuals for these two emotions. This effect was further modified by the presence of overt depressive status, Emotion × OC Intake × Subgroup interaction: *F*(4, 196) = 3.17, *p* = 0.015; *η*^2^_p_ = 0.061 (see Figure 3B). Participants with a high level of depressive symptoms (HiD) were slower than Non-HiD women, but also exhibited a larger effect of OC intake, particularly for the sad expressions (Figure 3B).

Perceived intensity of the emotions varied with OC intake. Repeated-measures ANOVA with Intake (Active, Inactive) and Emotion (Happy, Sad, Fear, Anger, Neutral, Disgust) as factors revealed that valenced facial expressions were perceived as more intense during active OC intake, main effect of OC Intake: *F*(1, 51) = 4.34, *p* = 0.042. The effect size was small to medium, Active: *M* = 6.42, *SD* = 0.81; Inactive: *M* = 6.27, *SD* = 0.81; *η*^2^_p_ = 0.078. Depressive status was found to moderate the effect of active intake. However, simple effects confirmed a significant OC intake effect even if Non-HiD were considered on their own, *F*(1, 36) = 8.51, *p* = 0.006.

### Affect Misattribution Procedure (AMP)

The classic AMP and Faces AMP were each analyzed using repeated-measures ANOVA, with OC Intake (Active, Inactive) and Type of Prime (Flower, Insect or else Happy, Sad or Angry) as factors. To test if depressive affect influenced the AMP, Subgroup was included as a between-subjects factor.

**Figure 4:**
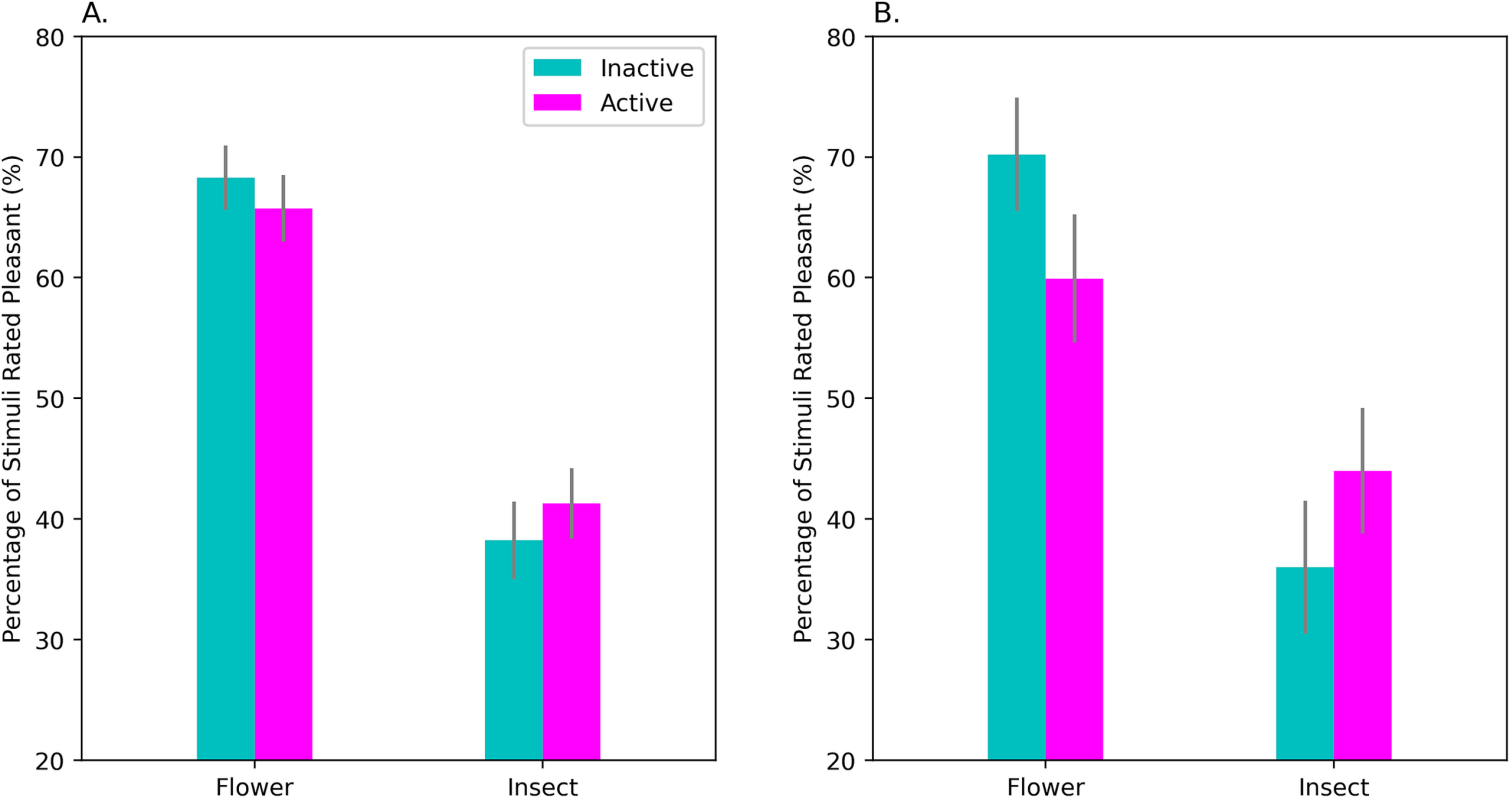
Performance on the classic AMP by OC-users in the sample as a whole **(A)** and in the HiD-Subgroup **(B)**. Shown on the *Y*-axis is the mean percentage of neutral characters rated as pleasant for each type of affective prime (flowers and ominous-looking insects). A classic priming effect was found. If flowers were used as primes, a high percentage of the succeeding characters were rated as pleasant, whereas insect primes elicited unpleasant ratings on a majority of the trials. Comparing performance during active (‘Active’) and inactive (‘Inactive’) OC intake revealed that affective priming was significantly weaker during the active intake of OC steroids. This effect was evident in the sample as a whole **(A)** but was largest for the HiD women **(B)** where a large effect of OC intake was observed. Note that the effect of an affective prime was weaker during active intake for both positively- and negatively-valenced primes. AMP, Affect Misattribution Procedure. Bars show the standard error of the mean.

Both AMP tasks showed the expected implicit priming effects (*p*’s < 0.001). Chinese characters immediately following a positive prime were more likely to be classified as “pleasant,” whereas targets that followed a negative prime were more likely to be classified as “unpleasant” (see Figure 4A). There was no significant effect of OC Intake on the priming effect for the Faces AMP. However, OC Intake did influence priming on the classic version of the AMP, both in the OC sample as a whole, *F*(1, 49) = 4.49, *p* = 0.039; *η*^2^_p_ = 0.084 (Figure 4A) and particularly in the HiD-subgroup, as revealed by simple effects analysis, *F*(1, 13) = 9.23, *p* = 0.010; *η*^2^_p_ = 0.415 (Figure 4B). Compared with no-intake, priming was visibly weaker during the active use of OCs.

Although the effect of OC intake was very prominent in the HiD women, no intake effect was seen in the Non-HiD group, if considered on its own, *F*(1, 36) = 0.160, *p* = 0.691; *η*^2^_p_ = 0.004. This suggests that the AMP effect was linked to the presence of depressive affect.

### Emotional Stroop

**Figure 5:**
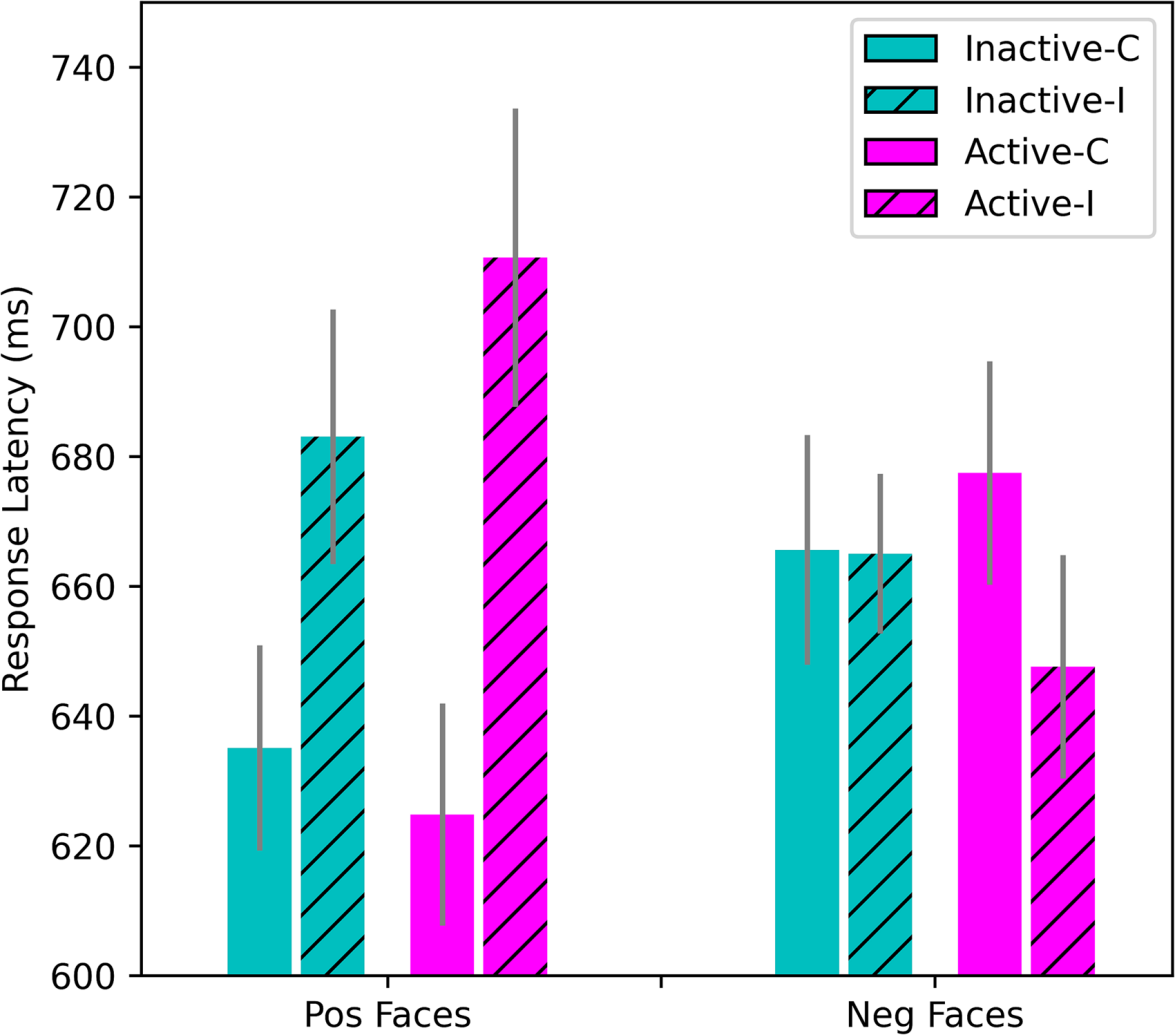
Effects of stimulus congruency and OC intake on responses to positively- and negatively-valenced faces on the Emotional Stroop. Hatched bars show the incongruent conditions and open bars the congruent. Only data for the HiD-subgroup are shown here (*N* = 15), but results for the OC sample as a whole were essentially the same. RTs for positive faces combined with positive words were the fastest, whereas negative faces elicited slower processing. For the positive faces, there was a strong Stroop interference effect under incongruent conditions (when a negative word was shown simultaneously). For the negative faces, there was little evidence of any additive slowing caused by the presence of a conflicting positive word.

On the Emotional Stroop, repeated-measures ANOVA of the RTs showed the expected interference effects (*p* < 0.001). Overall, RTs were slower on incongruent trials where the valence of the face and word were in conflict. RTs were also slower for negative than positive words overall. In the sample as a whole, the presence of a negative target word was associated with slowed responding on trials where positive faces were shown (*M* = 627 ms on congruent trials, *M* = 689 ms on incongruent trials where a negative word was present), while the presence of a positive word exerted little interference effect on RTs when sad/angry faces were shown (*M* = 663 ms on congruent trials, *M* = 652 ms on incongruent), *F*(1, 50) = 84.82, *p* < 0.001; *η*^2^_p_ = 0.629. This pattern was moderated by the presence of explicit depressive affect and OC intake, *F*(1, 50) = 12.69, *p* < 0.001; *η*^2^_p_ = 0.202. In the HiD-subgroup, shown in Figure 5, the effect of OC intake was statistically significant, *F*(1, 14) = 8.41, *p* = 0.012; *η*^2^_p_ = 0.375 (simple effects). Interference by negative words was stronger during active intake. If anything, a conflicting positive word shortened the RTs in the negative face condition during that time (Figure 5). In the Non-HiD subsample (with HiD removed), the OC intake effect was qualitatively similar but weaker, *F*(1, 36) = 4.04, *p* = 0.052; *η*^2^_p_ = 0.101 (data not shown in figure).

Accuracy of word classification was close to 100% in all conditions. Thus, individual differences in the present study were expressed primarily in the response times.

Results for the control task, the classic Color-Word Stroop, are shown in Supplementary material. The Color-Word Stroop was procedurally similar to the Emotional Stroop, but lacked any affective element in that only colors and words were presented. A significant interference effect was found on the incongruent trials (*p* < 0.001), but neither OC intake nor depression influenced performance.

## Discussion

In a group of healthy young women using conventional OCs, we observed a depression-like pattern of performance on implicit measures of mood during periods of active hormone intake. This result was driven partly, but not completely, by a subset of OC-users who acknowledged having elevated levels of negative affect. Effects were seen during active hormone use relative to the washout week of the contraceptive cycle when absence of hormone intake for as long as 7 days results in diminishing bodily concentrations of OC steroids (Edelman et al., 2014). The fact that a difference was visible over such a short timeframe suggests a neuroendocrine effect that is relatively short-term (although our study design cannot address if longer-term effects might also occur; cf. Anderl et al., 2020). The differences we observed were consistent and conformed to our *a priori* predictions. In contrast, the POMS (an explicit measure of ‘subjective’ mood), showed that participants perceived their own moods to be worse at the inactive not active phase of the contraceptive cycle.

To our knowledge, this is the first study to use implicit tasks to evaluate mood in OC users. Implicit measures have been used to study current or remitted depression in many other contexts (Payne and Lundberg, 2014). Compared with non-depressed individuals, people with major depression display differences on several implicit tests, including larger interference effects on the Emotional Stroop (Epp et al., 2012; Hu et al., 2012; Başgöze et al., 2015), altered decoding of facial signals including decreased attention to positive stimuli and decreased accuracy in identifying positive expressions as happy, greater attentional allocation to negative expressions (especially sad ones), are slower to react to facial expressions if RT is measured, and judge negative but not positive expressions as more intense than controls (Leppänen, 2006; Münkler et al., 2015; Weightman et al., 2014). All of these are reminiscent of the patterns we observed among the HiD in the present study. Our HiD-subgroup showed elevated Depression subscale scores on the POMS, indicating high negative affect by self-report. However, the POMS is a screening instrument only. Although it quantifies negative affect, it lacks items that evaluate the appetitive, cognitive, or motivational dimensions of clinical depression (American Psychiatric Association, 2013). Accordingly, we cannot conclude that our HiD-subgroup would meet criteria for clinical depression based on gold standard clinician interviews. However, the HiD-subgroup did display high negative affect that was very comparable in severity to norms for outpatients with clinically-ascertained mood conditions (McNair and Heuchert, 2010). They also performed on implicit tests like patients with verified depression. Importantly, while the implicit effects were largest for the HiD-group, a weaker version of the same patterns was evident in the Non-HiD subgroup as well (when the HiD were removed). This might reflect a gradient in mood status or milder negative affect among the Non-HiD women.

Our data support recent epidemiological studies which suggest that depressive changes do occur in a subset of women who opt to use hormonal contraception. For example, a prospective cohort study of over a million women based on the National Prescription Register of Denmark showed that women prescribed hormonal contraceptives subsequently required antidepressants at higher rates than non-users or were more likely to be diagnosed with clinical depression (Skovlund et al., 2016). Hormonal contraceptive use is also associated with an increased risk for attempted or completed suicides (Skovlund et al., 2018). Similarly, Anderl et al. (2020) found that adolescent OC use was associated with an increase in depression risk compared with never-users. The focus of such studies, however, has been depression of a clinical magnitude, which does not speak to lesser mood changes that might be present among OC-users more prevalently. Depressive changes and their time course in OC-users are important to document, whether clinical or subclinical in severity. This knowledge is especially relevant to prospective OC users and their healthcare providers in order to enable fully informed reproductive decision-making and contraceptive counseling, especially given the great importance of negative affect to women’s everyday quality of life.

Estimates of the percentage of OC-users who develop dysphoria are widely variable. This reflects differences across studies in how depression is defined, but also the impact of several moderator variables (e.g., personal past history of depression, choice of progestin, Bengtsdotter et al., 2018; Hampson, 2023; Joffe et al., 2003). In a double-blind, placebo-controlled trial, Lundin et al. (2017) reported that the proportion of women with new-onset subclinical depression during a 3-mo trial of OCs versus placebo was <10%, based on the MADRS scale (Montgomery-Åsberg Depression Rating Scale). Using the POMS, we previously found an incidence of about 7–8% who scored in the clinical range (Hampson, 2023). However, in the study by Lundin et al. (2017), 24% of women allocated to OC treatment had significant mood deterioration by their own self-reports, in agreement with the higher numbers based on self-report in the present study (also see Johansson et al., 2023). Both estimates fall within the ranges reported previously by other labs. Hampson (2023) also identified significantly higher POMS Depression scores among users of OCs that contained third- or fourth-generation progestins, as compared to first- or second-generation OC pills. The same trend could be identified in the present dataset (see Supplementary material). Depressive symptoms associated with the use of hormone-eluting intrauterine devices (IUDs) also have been described (Skovlund et al., 2016; Stenhammar et al., 2023; Zettermark et al., 2018), and may be dose-dependent (Roland et al., 2023). Because IUDs release a progestin but contain no estrogens, the IUD findings combined with our own past and present data implicate the progestin constituent of OCs as a potential driver of mood changes.

To further explore the possibility of depressive symptoms associated with the active use of OCs, we adopted innovative implicit techniques in the present work. Elevated scores on the POMS relative to normative data for age and sex, plus a depressive shift in scores on the implicit tasks (which can be sensitive to mood even when it is not explicitly acknowledged by self-report) suggest that depressive side-effects do occur among a subset of OC-users. In a small number of women, these symptoms may rise to the threshold of a clinical impairment. Ethinyl estradiol is a co-constituent (along with a progestin) of standard combined OC pills and may help to counteract adverse mood changes, given that high estradiol has been linked with positive mood states in other contexts (e.g., Backström et al., 1983; Hampson, 1990; see Hampson, 2002), but the dose of ethinyl estradiol in recently developed OCs is low (Dickey and Seymour, 2021), and perhaps not always sufficient to act as an effective counterweight.

Precisely which hormonally-sensitive brain regions underlie the observed effects on mood is not presently known. Functional brain imaging, notably studies using functional MRI, are beginning to identify differences in brain activity between hormonal contraceptive users and non-users in resting-state activity, functional connectivity, or BOLD activation evoked during imaging tasks designed to elicit affective processing. These include decreased activity in OC users in the inferior frontal cortex, amygdala, insula; reduced amygdala reactivity to emotionally-charged images; and alterations in reward-related processing in response to monetary incentives, food, or erotic stimuli (for review see Brønnick et al., 2020; Pletzer et al., 2023). In a placebo-controlled trial, Hidalgo-Lopez et al. (2023) found connectivity changes during OC use in 2 forebrain networks that were predictive of adverse PMS-like mood effects. Differences in the brain regions that underlie implicit and explicit affect are thought to exist (e.g., DeCoster et al., 2006; Lane, 2008), but it is unknown how these might map onto the differences now being described between OC users and non-users in brain activation during emotional processing. Structural brain changes have also been reported in OC users, but evidence is still sparse and must be viewed as tentative (e.g., Lisofsky et al., 2016; Brouillard et al., 2023; for a critical review see Brønnick et al., 2020). Given that several of the regions implicated in OC studies participate more generally in emotional regulation, social cognition, or the subjective experience of emotion, and are also sites where progesterone receptors have been identified in animal studies (Sundström-Poromaa et al., 2020), they are possible candidate regions where OC steroids might exert neuroregulatory actions to bring about alterations in mood. Further research will be important to help illuminate the pathways concerned.

In their natural form, sex steroids exert a wide spectrum of cellular and molecular effects in the CNS, that operate at various time scales. Modifications in neurogenesis, synaptogenesis, myelination, and neurotransmitter signaling have been reported in animal studies (see Barth et al., 2015; Pletzer et al., 2023 for recent reviews). Estrogen- or progesterone-mediated effects in serotonergic, dopaminergic, noradrenergic, GABA, and several other brain systems (e.g., McEwen and Alves, 1999) are of special interest, as are hormone-mediated effects on regional synapse densities (Woolley and McEwen, 1993), because they occur in the adult CNS, vary dynamically with hormone availability in the bloodstream, and occur rapidly, within minutes, hours, or days. Their time course makes them attractive prospects to explain active versus inactive OC phase differences. It should be noted that contraceptive steroids have received little study in a neurobiological context (Pletzer et al., 2023). However, many of the effects seen for the natural forms of the steroids are likely shared by contraceptive steroids, to the extent that they can occupy the same hormonal receptors in the CNS. The situation in OC users is complex, however, because some synthetic progestins used in OCs possess a wider hormone receptor binding profile than the natural form of progesterone (Dickey and Seymour, 2021; Hampson, 2023; Sitruk-Ware, 2006), may alter the production of neuroactive steroids (Pletzer et al., 2023; Rapkin et al., 2006), and also impact the HPA (hypothalamic–pituitary–adrenal) axis, stress reactivity, and immune system responses (e.g., Kirschbaum et al., 1999), all of which may contribute to mood. The monoamines are especially important for the regulation of mood and depression. One recent study showed a ~10% lower brain serotonin receptor-4 binding potential (5-HT4R) in OC users (Larsen et al., 2020; but see Larsen et al., 2022). An advantage of the active vs. inactive study design used here is its potential to narrow the range of potential mechanisms by pointing to brain effects that are acute, relatively short-acting, and reversible.

A surprising aspect of the present work was that while our implicit tasks suggested negative mood was greater during the active use of exogenous hormones, consistent with our *a priori* theoretical predictions, our *explicit* measure of mood, the POMS, suggested that self-perceived negative affect was greater during the inactive (‘washout’) phase. During the inactive portion of the contraceptive cycle, a dropoff occurs in serum concentrations of the exogenous steroids, which triggers shedding of the uterine lining in the form of menstrual flow (Edelman et al., 2014). We observed that POMS Depression scores were elevated in our HiD participants (and to some extent the Non-HiD) at both phases of the contraceptive cycle, and therefore the inferences drawn above apply. Nevertheless, a lack of direct parallelism between the implicit and explicit effects was unexpected (even though the psychological literature shows that dissociations can occur in certain situations, consistent with separability in the neural circuitry that underlies implicit and explicit emotional responses; DeCoster et al., 2006; Suslow et al., 2019). Our study allows rare insight into the explicit mood changes in OC-users, as past studies of the impact of OCs have understandably limited their assessments mostly to periods of active hormone usage. However, our data reinforce sporadic research reports indicating that self-perceptions of negative affect might in fact be higher during the inactive phase of the contraceptive cycle (Noachtar et al., 2023). Future studies will be needed to clarify this finding.

It is unclear why our implicit and explicit measures did not change in a more synchronized fashion, but explicit ratings of negative affect can demonstrate rapid change in other contexts (e.g., under a short-term course of psychotherapy, Suslow et al., 2019) and are subject to top-down control from other cortical inputs that leave implicit responses unaffected, such as conscious verbal representations or inputs from declarative memory systems. In general, a differentiation between implicit and explicit affect is theoretically valuable because they are thought to stem from two interacting but independently-operating emotional processing networks—an automatic/autonomic system and a reflective system that operates within conscious control. Implicit affect can predict individuals’ behavioral and psychophysiological reactions to stress or emotional stimuli (e.g., cortisol or cardiovascular reactivity) either exclusively or beyond the predictive value of explicit measures alone (Suslow et al., 2019). Independent of any physiological driver, conscious self-estimations of mood may be more malleable than implicit measures are (DeCoster et al., 2006; Suslow et al., 2019) and thus perhaps more subject to day-to-day variation in subjective perceptions, attentional biases, self-presentation concerns, the demand characteristics inherent in a research setting, or societal expectations based on traditional stereotypes regarding mood and the menstrual cycle or personal past history. It is also possible that other kinds of interacting physiological variables, such as cycle-related variations in sleep quality, exert a larger temporary influence on explicit ratings during the inactive than active phase (even though somatic and appetitive effects tend to be milder and less prominent under OC use; Sundström Poromaa and Segebladh, 2012). If true, then implicit techniques might offer a more stable and perhaps accurate window into changes in underlying neurophysiology related to primitive affective processes.

In non-OC users, self-reported negative affect among a susceptible group of women is most clearly identified with the premenstrual days of the ovarian cycle. At that time, serum concentrations of estradiol and progesterone transition from the highs of the preceding midluteal phase to the lows of the menses that follow (Steiner et al., 2003). Although temporally displaced, certain endocrine parallels exist between the premenstrual phase of the natural menstrual cycle and the no-intake phase of the OC cycle, as intake of exogenous steroids in OC-users is discontinued abruptly at the end of the active phase. Furthermore, evidence implicates progestogens or, more particularly, progestogen withdrawal, in both types of mood phenomena (for a review see Sundström-Poromaa et al., 2020). The timing of the self-perceived negative mood in OC users may also become more comprehensible given that ethinyl estradiol in the bloodstream is metabolized and reaches low steady-state values more rapidly at the beginning of the inactive phase than progestin, leaving unopposed residual progestin activity for 2–3 days at the onset of the inactive interval (Edelman et al., 2014). Mood lability and vulnerability to negative affect occur during other hormonal transition points in the female lifecycle too, such as parturition or the perimenopausal transition (Gordon et al., 2018; for review see Steiner et al., 2003), adding further plausibility to the possibility that mood changes might occur in a subset of users in response to OC transitions. Interestingly, there are also data to suggest that women who are susceptible to major depression may experience larger self-perceived mood effects than non-susceptible women under the influence of ovarian steroids (e.g., Bloch et al., 2000; Sundström-Poromaa et al., 2020), including OCs (Oinonen and Mazmanian, 2002). This might help explain why intake-related mood effects were larger for the HiD-group in the present study.

The present study is the first to demonstrate depressive-like effects on implicit measures of mood in OC users. On implicit tests, depression reveals itself without a respondent’s awareness, in the form of objective differences in performance on automatic measures of attentional or perceptual bias, affective reactions, or valenced emotional processing. The potential for desynchronization of implicit and explicit measures highlights the complexity of affective processing in general, and the importance of using several different types of measures to assess affective processing. Inclusion of implicit tasks may prove useful in future studies of OCs and their possible mood effects. These might include the AMP (Payne et al., 2005; Murphy and Zajonc, 1993), which has been widely used in many other contexts. Although the classic version of the AMP was successful in the present study, our use of emotional faces as priming stimuli was not. This was due to our unfortunate decision to use an identical exposure time, which was adequate for simple flower and insect images but turned out to be inadequate for participants to apprehend the greater visual complexity of the emotional expressions we chose as primes (cf. LeMoult et al., 2012, who used a 500 ms prime exposure time). As a result, the face version of our AMP task significantly underperformed. A longer prime exposure is therefore recommended for future studies if faces are to be used as primes.

The present work was intended as a pilot-level study. It will thus be helpful for future larger-scale investigations to recruit a greater sample size. Among other things, this would enable comparisons to be made among the different families of contraceptive progestins. This could help determine if any differences exist in their potential for adverse mood effects. Studying implicit mood in non-OC users would likewise be useful, to allow direct comparisons between OC-users and non-users. In a previous study done by our laboratory (Hampson, 2023), non-users were in fact assessed. Relative to non-users, POMS Depression subscale scores were found to be significantly higher among a group of healthy women taking OCs (*N* = 193). Careful thought will need to be given to recruitment methods and which stages of the menstrual cycle to target if making mood comparisons between OC users and non-users in future studies. While the current study used a prospective design, which is desirable, we were not able to control the length of time post-OC initiation when mood status in our sample was evaluated. If we assume that OC-users who encounter troublesome mood side-effects following the initiation of OC use are more likely to discontinue taking their OCs, the present study might under-estimate the percentage of women who experience depressive changes as a function of OC use due to differential dropout over time. On the other hand, it is a strength of the present work that the median number of months on the OC pill in the current study was 12 months, as some evidence suggests mood effects are seen most frequently during the first 2 years of OC usage (Johansson et al., 2023). Other strengths of the present work are the enrollment of medication-free participants, which avoids confounds introduced by other concurrent medication use or pre-existing health conditions that might otherwise create ambiguities in attributing depressive symptoms to OC use *per se*. On the other hand, in clinical practice OCs are often prescribed under less pristine conditions to women who have other complex health conditions that may affect OC outcomes such as a personal history of affective disorder (Bengtsdotter et al., 2018; Oinonen and Mazmanian, 2002). This means that effect sizes seen for associations between OC use and implicit measures of depressed mood might potentially be even larger under real-world conditions. Of course, a final but significant strength of the present study is its inclusion of implicit tests instead of the usual exclusive reliance on self-report to detect depressive symptoms.

The present data provide novel insights into affective processing in women who use OCs. Our work converges with other recent investigations which suggest that endocrine states are surprisingly relevant to social cognition. Effects of the menstrual cycle on women’s evaluations of men’s sexual attractiveness have been reported, as have effects of testosterone on men’s aggressive responses but also self-initiated acts of generosity and trust toward others (Boksem et al., 2013; Dreher et al., 2016; Penton-Voak and Perrett, 2000). The ability of sex steroids to modulate brain pathways involved in emotion makes them particularly powerful regulators of human behavior. Because the implicit tasks used in the present study required accurate emotional decoding of faces (e.g., the FEIT, Emotional Stroop), they further illustrate the subtle impact of an ostensibly neutral endocrine variable—oral contraceptive use—on social appraisals, social functioning, and the interpersonal environment, in this case mediated by depressive brain changes linked to the use of OCs. Successful processing of facial signals is vital to the ability of a ‘receiver’ to generate accurate emotional appraisals and well-calibrated responses to social cues, in turn helping to maintain the quality of personal relationships and close emotional bonds with loved ones (Fischer and Manstead, 2016). Any effects of OC steroids on social cognition are therefore of potential consequence to the millions of women who choose a hormonal form of contraception.

## Data Availability

The datasets presented in this study can be found in online repositories. The names of the repository/repositories and accession number(s) are: Open Science Framework (OSF), http://doi.org/10.17605/OSF.IO/WCRN4.
