# Supplementary material for: Current oral contraceptive use affects explicit and implicit measures of depression in women
Source: Front Psychol. 2024 Oct 18;15:1462891. doi: 10.3389/fpsyg.2024.1462891 (PMC11527683; doi:10.3389/fpsyg.2024.1462891)
Supplement: Supplementary file 1 [file Table_1.DOCX]

**Supplementary Materials**

**A. Details of Procedures for the Computerized Tasks**

**1. Affect Misattribution Procedure (AMP)**

Source: Millisecond Software LLC, Inquisit Lab 5

Procedure: 1 practice block of 10 trials

1 test block of 48 trials

Exposure time for priming stimulus: 75 ms

Prime is followed by blank ISI: 125 ms

Exposure time for target: 100 ms

Exposure time for visual mask: until keyboard response is made

How does participant respond: keyboard response (buttonpress) using designated keys

Notes:

We used the standard set of flower and insect images, and the standard Chinese character set from the Inquisit implementation of the AMP. From an available pool of 200 Chinese characters, 48 images were selected at random each time the AMP script was run and served as the target stimuli. Therefore, the character set that was used was randomized between the participants, and between the classic AMP and face version of the AMP within participants.

All of the above details apply to the **classic flower-insect version** of the AMP.

For the **novel face version of the AMP**, devised for the present study, exposure times and stimuli were identical to those stated above, except that the flower and insect primes were removed and instead emotional face images were substituted as the positive and negative primes. Faces were selected from the RADIATE database of emotional faces (Conley et al., 2018), and consisted of equal numbers of happy faces (used as positive affective primes) and sad or angry faces (used as negative affective primes). Order of presentation of the emotional expressions was randomized. All faces were of ordinary attractiveness, depicted ‘everyday’ people, and were full frontal headshots photographed in color. Both open- and closed-mouth expressions were used as primes.

**2. Facial Emotion Identification Task (FEIT)**

Source: Labmade task designed for the current study, implemented in E-Prime 3.0

(Psychology Software Tools, Sharpsburg, PA).

Procedure: 1 practice block of 4 trials

1 test block of 96 trials

Exposure time for target face: 10 sec (max, if not terminated earlier by buttonpress)

Exposure time for visual mask: 20 ms

Exposure time for intensity rating: unlimited

How does participant respond: keyboard response (buttonpress) using designated key

Notes:

Images of adult emotional faces were selected from the Pictures of Facial Affect (Ekman & Friesen, 1976) and the RADIATE dataset of emotional faces (Conley et al., 2018; Tottenham et al., 2009). Equal numbers of male and female faces were used. Targets were presented in randomized order, and were displayed at eye level on a 30 x 54 cm rectangular flatscreen monitor at a distance of approximately 50-60 cm. The participant sat directly in front of the screen. Response latency on each trial was recorded in milliseconds by E-Prime and was calculated as the time elapsed from the moment of image onset to onset of the participant’s keypress response. A keypress terminated the face image and initiated a visual mask (an abstract greyscale image used to terminate visual processing). After 20 ms the mask disappeared and a secondary screen was displayed, containing a 10-point Likert response scale, shown on a horizontal line, ranging from 1 (“Not at all intense”) to 10 (“Extremely intense”). The participant used the response scale (via a vocal response) to rate the perceived emotional intensity of the facial expression they’d just viewed.

The following six emotions were used to test the speed and accuracy of emotion identification:

Happy Neutral or calm

Sad Fear

Angry Disgust

The 96 emotional faces (“targets”) were presented in an individually randomized order. Participants were instructed to identify the emotion that was portrayed on each trial, and were asked to respond as quickly as possible.

**3. Emotional Stroop**

Source: Labmade task designed for the current study, implemented in E-Prime 3.0

(Psychology Software Tools, Sharpsburg, PA). The facial stimuli were full-color images selected from the RADIATE emotional faces set.

Procedure: 1 practice block of 10 trials

1 test block of 128 trials

Exposure time for the stimuli: 1000 ms (max, if not terminated earlier by buttonpress)

Exposure for interstimulus screen: 1000 ms

How does participant respond: keyboard response (buttonpress) using designated keys

Notes: Stimuli were shown on a flat-screen desktop monitor and were approximately life-sized. Sixty-four face images were used. They displayed angry, happy, sad, or neutral expressions. For each category of emotion, an equal number of male and female faces was shown. Each face was presented twice, once in a congruent trial (the face was paired with a word having the same emotional valence, such as a smiling face paired with the word “love”), and once in an incongruent trial (the face was paired with a word that did not have the same emotional valence, such as a sad face paired with the word “sunshine”). Neutral faces were also shown twice.

Each of the 128 test trials (and 10 practice trials) involved the presentation of a compound stimulus composed of an emotional face on which an emotionally-laden word was superimposed. The word was positioned in the foreground of the face, at approximately mid-face level (just beneath the eyes). All words were printed in a plain blue 60-point lowercase font. The order of the test stimuli was randomized. Participants were instructed to press one of two keys on the computer keyboard to indicate on each trial if the word shown was positive or negative (while ignoring the valence of the face that was simultaneously displayed). The “1” key was pressed to indicate a positively valenced word, and the “2” key to indicate a negatively valenced word. Participants were told to respond as quickly as possible. A keypress immediately terminated the stimulus image and caused a central fixation cross (+) to appear on the screen until the next face appeared. If no keypress was received, the stimulus screen terminated after 1000 ms, but a keypress response continued to be permitted throughout the interstimulus interval (of 1000 ms), which therefore allowed 2000 ms in total for participants to make their response on each trial.

The dependent variables recorded by E-Prime on each trial were accuracy (of the participant’s word classification) and response time (in milliseconds).

Our implementation of the Emotional Stroop was modelled on a task used previously by Basgõze et al. (2015). English instead of Turkish words were used as stimuli in the current study.

**B. Influence of Progestin Subtypes**

A large number of different progestins are used in contemporary oral contraceptive formulations. These fall into 4 distinct family clusters that are defined by differences in the chemical structure of the progestins (see Dickey & Seymour, 2021; Hampson et al., 2022). The biochemical differences confer differences in progestogenic potency, but also in the extent to which the progestins possess the ability to bind to other classes of steroid receptors, e.g., androgen receptors.

To explore if the type of progestin contained in an OC pill might be relevant to its association with mood effects, we classified each brand of pill used in the present study according to the family of progestin it contained. We then ran an ANOVA to compare mean scores on the POMS Depression subscale across the resulting progestin families. Classification of the individual progestins was based on recognized pharmaceutical sources (Dickey & Seymour, 2021; Sitruk-Ware et al., 2013).

Classification into progestin families resulted in the following numbers:

| Progestin Generation (Family) | Names of Individual Progestins Represented | Number in Current Sample |
| --- | --- | --- |
|  |  |  |
| 1 | Norethindrone acetate | N = 4 |
|  |  |  |
| 2 | Levonorgestrel | N = 26 |
|  |  |  |
| 3 | Norgestimate or desogestrel | N = 21 |
|  |  |  |
| 4 | Drospirenone or cyproterone^#^ | N = 11 |

^#^ Cyproterone is an older synthetic progestin and is sometimes considered first generation. It is classified here with drospirenone because both are potent progestogens which differ from the other families in having strong anti-androgenic properties.

Total N = 62

Generation 1 progestins could not be included in the ANOVA, because there was only 1 woman using a first-generation progestin once the 3 participants using Lolo were excluded. Because the number of fourth-generation contraceptives was still very limited (N = 11), we combined the third- and fourth-generation progestins into a single group for purposes of statistical analysis. The decision to combine was based on an earlier dataset from our laboratory (Hampson, 2023), where we observed that negative affect as revealed by the POMS was elevated similarly amongst women taking either third- or fourth-generation pills (see Fig. 2 in Hampson, 2023).

We then ran a two-way ANOVA with Type of Progestin (Gen2, Gen3/4) as a between-subjects factor and Intake Status (Active, Inactive) as a repeated-measures factor. Each participant’s self-ratings of negative affect on the POMS Depression subscale during Active and Inactive intake was analyzed as a dependent variable. As stated in the Results, log-transformed POMS scores were analyzed but the raw score means are shown in Supplementary Fig. 1 for simplicity.


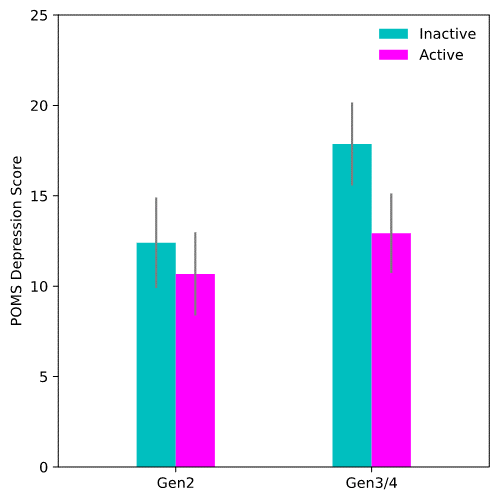


**Supplementary Fig. 1.** Mean POMS Depression scores for women using OCs that contained a second-generation progestin (Gen2) versus a third- or fourth-generation progestin (Gen3/4). Data are shown separately for active OC pill intake (‘Active’) and for the no-intake condition (‘Inactive’). Depression scores tended to be higher for users of third- or fourth-generation pills than second-generation pills, but the main effect of progestin subtype did not reach the *p* < .05 threshold for significance. Decomposition of the interaction between intake and progestin subtype revealed no significant intake effect among women using second-generation pills (*p* = .359), but women using OCs containing a third- or fourth-generation progestin exhibited a significant difference in POMS scores as a function of intake (*p* = .006). Self-reported depression was higher during the no-intake interval (inactive portion of the contraceptive cycle). Pairwise comparisons were done using the least significant difference method. Error bars depict the standard error of the mean.

It will be essential for future studies to confirm this difference with a larger sample of OC users, as the current sample was not adequately powered to test for differences among progestin subtypes.

**C. Color-Word Stroop (Control)**

Source: Millisecond Software LLC, Inquisit Lab 5

Procedure: 1 test block consisting of 84 trials

Exposure time for the stimuli: until a keypress response is made

Intertrial interval: 200 ms

Error feedback screen: 400 ms

How does participant respond: keyboard response (buttonpress) using designated keys

Notes: The classic color-word Stroop test (Jensen & Rohwer, 1966) was administered as a control task, using a standard Inquisit script with keyboard responding. On each trial, a printed word (or a rectangular color patch) was displayed in the center of the computer screen (e.g. “green”, “blue”, “red”, “black”). On ‘congruent’ trials, the color of the font in which the word was printed matched the meaning of the word displayed (e.g. the word “red” shown in a red font). On ‘incongruent’ trials, the color of the font did not match the semantic meaning of the word. Participants were asked to respond as quickly and accurately as possible whenever a stimulus appeared by pressing a key to indicate the color in which the stimulus was displayed, while ignoring the semantic content of the words. Stimuli were presented on a flat-screen desktop monitor. Congruent and incongruent trials were presented in an intermixed, randomized order.

The dependent variables recorded by Inquisit on each trial were accuracy (of the participant’s response) and response latency (in ms).

The Color-Word Stroop was analyzed using a mixed factorial ANOVA with Intake (Active, Inactive) and Congruency (Congruent, Incongruent) as within-subjects factors and Depression Status (HiD, Non-HiD) as a between-subjects factor. Mean latency (RT) to generate a correct response was the dependent variable.

Means and SDs are shown in the following table:

| **Descriptive Statistics** | | | | |
| --- | --- | --- | --- | --- |
|  | DEPRESSION_BINARY | Mean | Std. Deviation | N |
|  |  |  |  |  |
| IA_STROOP_CONG_MSEC | .00 | 848.4163 | 182.67896 | 38 |
|  | 1.00 | 852.1341 | 169.43486 | 15 |
|  | Total | 849.4685 | 177.41804 | 53 |
| IA_STROOP_INCONG_MSEC | .00 | 1090.0955 | 310.55532 | 38 |
|  | 1.00 | 1026.6273 | 256.61254 | 15 |
|  | Total | 1072.1328 | 295.27277 | 53 |
| A_STROOP_CONG_MSEC | .00 | 860.8218 | 151.14398 | 38 |
|  | 1.00 | 944.2333 | 241.49818 | 15 |
|  | Total | 884.4289 | 182.74465 | 53 |
| A_STROOP_INCONG_MSEC | .00 | 1060.6350 | 252.65802 | 38 |
|  | 1.00 | 1119.6265 | 355.82695 | 15 |
|  | Total | 1077.3307 | 283.24835 | 53 |

The ANOVA results showed a significant Stroop interference effect, as evidenced by slower RTs on the incongruent than congruent trials, *F* (1, 51) = 78.40, *p* < .001. Though it was not significant, the mean RTs tended to be very slightly slower overall during active hormone intake, *F* (1, 51) = 2.09, *p* = .155, which was mainly attributable to the HiD subgroup, *F* (1, 51) = 3.02, *p* = .088. However, there was no main effect of depression (*p* = .740) and there were no significant interactions that involved depressive status (all *p*’s > .300). In particular, the effect of OC Intake on the congruency effect did not vary as a function of depressive status, *F* (1, 51) = 0.44, *p* = .508.

**D. Distributions of Response Times (RT) on the FEIT and Emotional Stroop**

**Facial Emotion Identification Task (FEIT)**


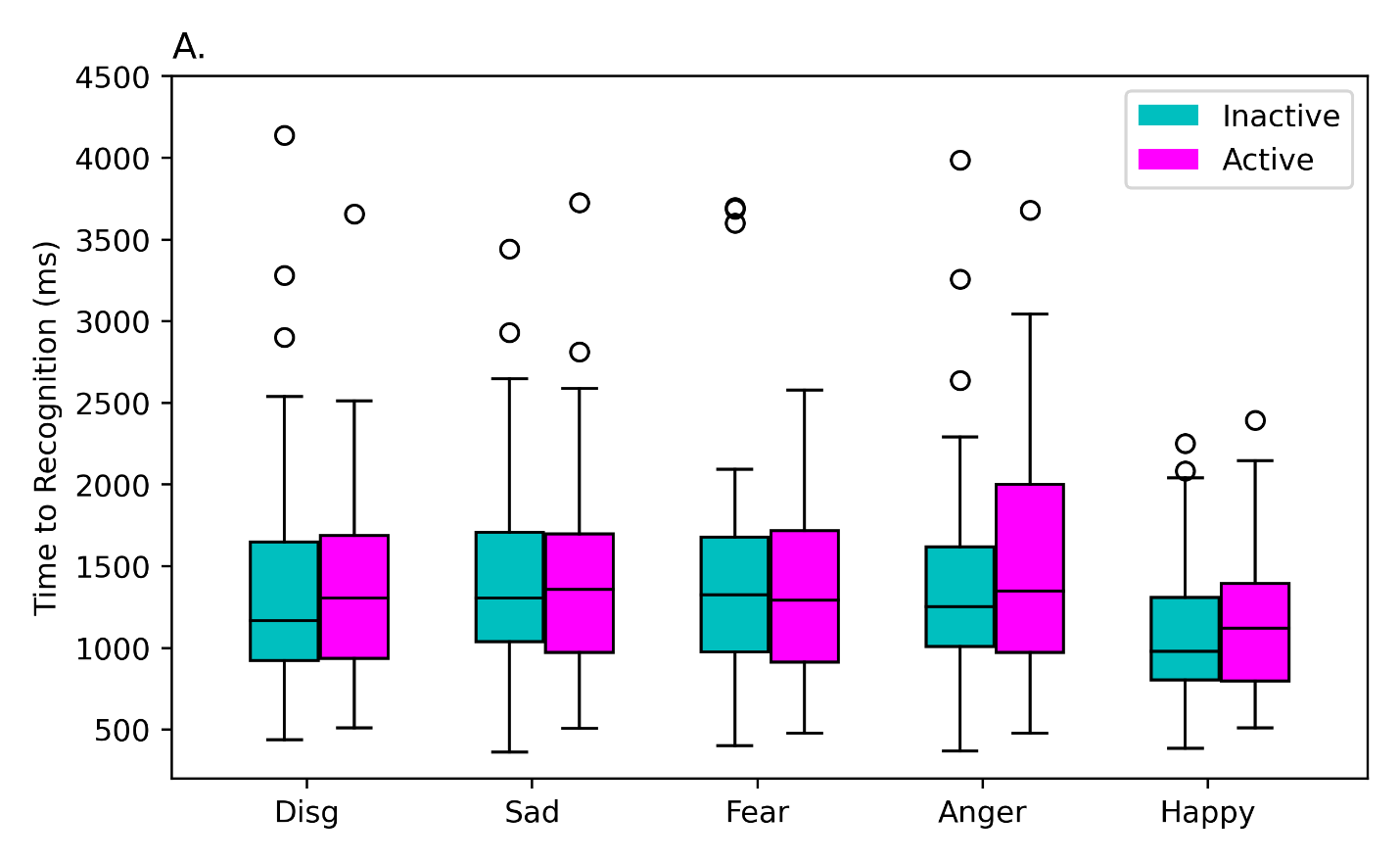


Note**:**  Disg = Disgust, other emotions are as labelled. Labels denote the category of facial emotion depicted by the facial stimuli that were presented. A within-subjects design was used, so the same individuals were tested at both the Active and Inactive phases of oral contraceptive (OC) intake, *N* = 53. Data shown here include outliers.

**Emotional Stroop**


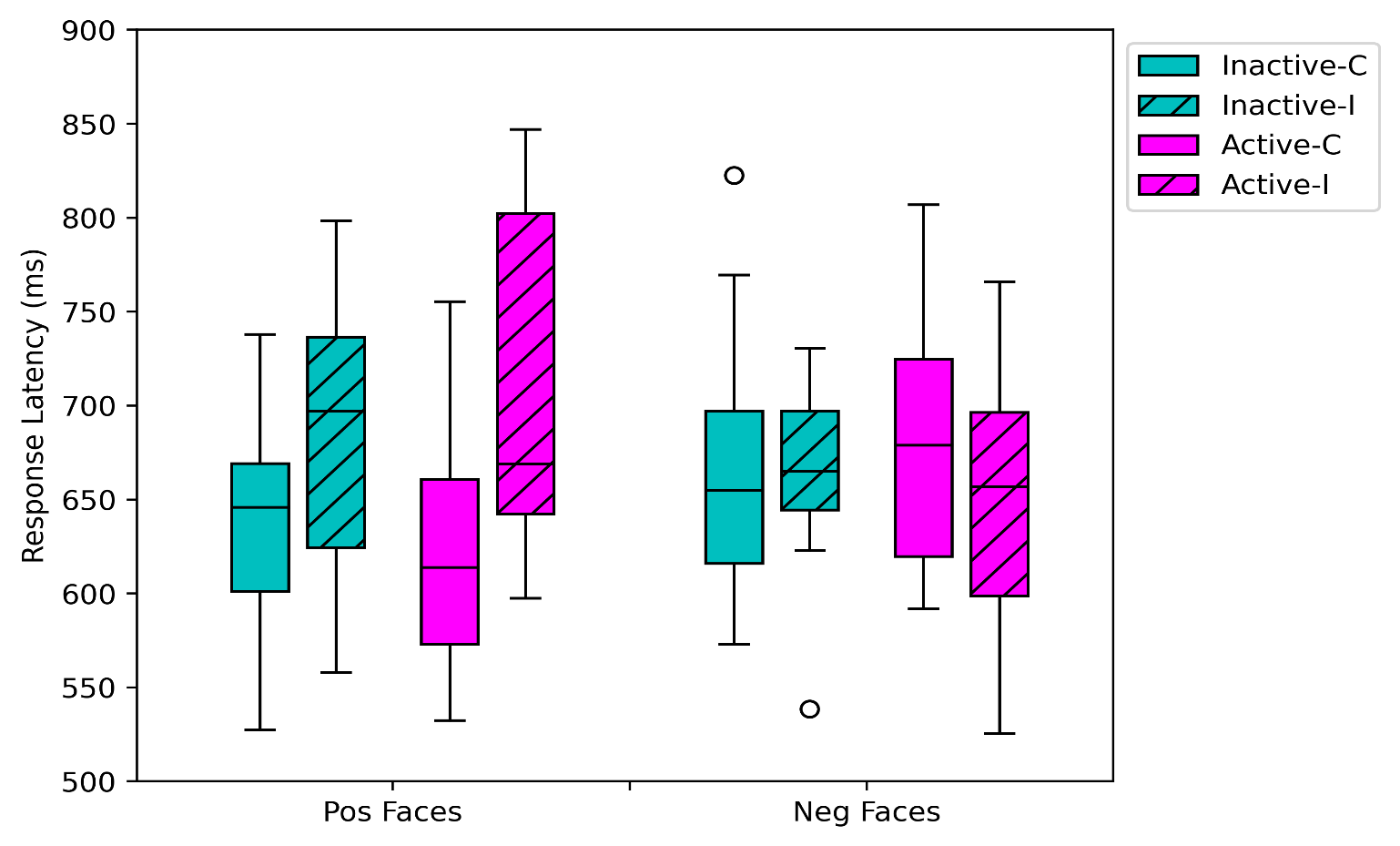


Note**:**  Pos Faces = Positive Faces, Neg Faces = Negative Faces. In the legend, “C” denotes the conditions where the word stimuli were affectively congruent with the stimulus face shown on each trial and “I” denotes the conditions where the words were affectively incongruent with the face. A within-subjects design was used, therefore the same individuals are represented in the figure at both the Active and Inactive phases of oral contraceptive (OC) intake. Data shown are for the HiD Subgroup only (*N* = 15) who self-reported high levels of negative affect on the Profile of Mood States (POMS).

**E. References Cited in Supplementary Materials**

Başgöze, Z., Gönül, A. S., Baskak, B., & Gökçay, D. (2015). Valence-based Word-Face Stroop task reveals differential emotional interference in patients with major depression. *Psychiatry Research*, 229, 960–967. <https://doi.org/10.1016/j.psychres.2015.05.099>

Conley, M. I., Dellarco, D. V., Rubien-Thomas, E., Cohen, A. O., Cervera, A., Tottenham, N., Casey, B. J. (2018). The racially diverse affective expression (RADIATE) face stimulus set. *Psychiatry Research*, 270, 1059-1067. <https://doi.org/10.1016/j.psychres.2018.04.066>

Dickey RP, Seymour ML. (2021). *Managing Contraceptive Pill Patients and Other Hormonal Contraceptives* (17th ed). Fort Collins, CO: EMIS Medical Publishers.

Ekman, P., Friesen, W. V. (1976). *Pictures of Facial Affect*. Palo Alto, CA: Consulting Psychologists Press.

Hampson, E. (2023). Oral contraceptives in the central nervous system: Basic pharmacology, methodological considerations, and current state of the field. *Frontiers in Neuroendocrinology*, 68, 101040. doi:10.1016/j.yfrne.2022.101040

Hampson, E., Morley, E. E., Evans, K. L., Fleury, C. (2022). Effects of oral contraceptives on spatial cognition depend on pharmacological properties and phase of the contraceptive cycle. *Frontiers in Endocrinology*, 13, 888510. doi: 10.3389/fendo.2022.888510

Jensen, A. R., Rohwer, W. D. (1966). The Stroop color-word test: a review*. Acta Psychologica,* 25, 36–93.

Sitruk-Ware R. (2006). New progestogens for contraceptive use. *Human Reproduction Update* 12(2), 169–78. doi: 10.1093/humupd/dmi046

Tottenham, N., Tanaka, J. W., Leon, A. C. McCarry, T., Nurse, M., Hare, T. A. et al. (2009). The NimStim set of facial expressions: Judgments from untrained research participants. *Psychiatry Research*, 168, 242-249. doi: 10.1016/j.psychres.2008.05.006
